# Evaluation of the dosimetric and radiobiological parameters in four radiotherapy regimens for synchronous bilateral breast cancer

**DOI:** 10.1002/acm2.13706

**Published:** 2022-06-21

**Authors:** Sang‐Won Kang, Seonghee Kang, Boram Lee, Changhoon Song, Keun‐Yong Eom, Bum‐Sup Jang, In Ah Kim, Jae‐Sung Kim, Woong Cho, Dong‐Suk Shin, Jin‐Young Kim, Jin‐Beom Chung

**Affiliations:** ^1^ Department of Radiation Oncology Seoul National University Bundang Hospital Seongnam‐si Gyeonggi‐do Republic of Korea; ^2^ Department of Radiation Oncology Seoul National University Hospital Seoul Republic of Korea; ^3^ Department of Radiation Oncology Seoul National University Boramae Medical Center Seoul Republic of Korea; ^4^ Proton Therapy Center National Cancer Center Goyang‐si Gyeonggi‐do Republic of Korea; ^5^ Departments of Radiation Oncology Dongnam Institute of Radiological and Medical Sciences Busan Republic of Korea

**Keywords:** dosimetric parameters, intensity‐modulated radiotherapy, radiobiological parameters, synchronous bilateral breast cancer, volumetric modulated arc therapy

## Abstract

This study is to investigate the optimal treatment option for synchronous bilateral breast cancer (SBBC) by comparing dosimetric and radiobiological parameters of intensity‐modulated radiotherapy (IMRT) and volumetric modulated arc therapy (VMAT) plans using single and dual isocenters.

Twenty patients with SBBC without lymph node involvement were selected retrospectively. Four treatment plans were generated for each patient using the Eclipse treatment planning system (Varian Medical System, Palo Alto, CA, USA) following two delivery techniques with two isocenter conditions—IMRT using a single isocenter (IMRT_Iso1), VMAT using a single isocenter (VMAT_Iso1), IMRT using dual isocenters (IMRT_Iso2), and VMAT using dual isocenters (VMAT_Iso2). A dose of 42.56 Gy in 16 fractions was prescribed for the planning target volume (PTV). All plans were calculated using the Acuros XB algorithm and a photon optimizer for a 6‐MV beam of a Vital Beam linear accelerator. PTV‐related dosimetric parameters were analyzed. Further, the homogeneity index, conformity index, and conformation number were computed to evaluate plan quality. Dosimetric parameters were also measured for the organs at risk (OARs). In addition, the equivalent uniform dose corresponding to an equivalent dose related to a reference of 2 Gy per fraction, the tumor control probability, and the normal tissue complication probability were calculated based on the dose–volume histogram to investigate the radiobiological impact on PTV and OARs.

IMRT_Iso1 exhibited similar target coverage and a certain degree of dosimetric improvement in OAR sparing compared to the other techniques. It also exhibited some radiobiological improvement, albeit insignificant. Although IMRT_Iso1 significantly increased monitor unit compared to VMAT_Iso1, which is the best option in terms of delivery efficiency, there was only a 22% increase in delivery time. Therefore, in conclusion, IMRT_Iso1, the complete treatment of which can be completed using a single setup, is the most effective method for treating SBBC.

## 1 INTRODUCTION

Breast cancer is one of the most common cancers in women, and many patients are diagnosed with breast cancer every year.^1^ Approximately 2% of all patients with breast cancer are diagnosed with synchronous bilateral breast cancer (SBBC).^2^, ^3^ SBBC, including two or more malignant tumors of the bilateral breast, is rare and complex, but the number of SBBC diagnoses is increasing steadily with the increase in breast cancer cases. Further, it is more common in younger patients and is characterized by tumors of a smaller size than those related to unilateral breast cancer (UBC).^4^

Radiotherapy (RT) planning for SBBC treatment is more challenging than RT planning for UBC. The difficulty of formulating a treatment plan is compounded if the planning target volume (PTV) includes axillary lymph nodes and/or internal mammary gland nodes.^3^, ^4^ In such cases, organs at risk (OARs) cannot be spared adequately in the direction of the PTV if traditional half‐field RT techniques, for example, 2D RT or 3D conformal RT (3DCRT) with tangential beam irradiation, are used.^5^, ^6^ Further, the application of these techniques on both sides may result in a high maxima above the sternum, where the tangential fields overlap and produce unacceptably low target coverage.^7^

Recently, intensity‐modulated radiotherapy (IMRT) and volumetric modulated arc therapy (VMAT) have been implemented clinically as treatment options to improve dose homogeneity and decrease normal tissue irradiation for complex treatment volumes, as in the case of SBBC.^7^, ^8^, ^9^, ^10^, ^11^ Nicolini et al. reported a planning study for VMAT and fixed‐field IMRT of 10 SBBC patients.^8^ They found that the VMAT technique, with simultaneous integrated boost, improved dosimetry and shortened treatment time compared to IMRT. Seppala et al. also reported that VMAT was superior to conventional tangential half‐field techniques in terms of PTV coverage.^7^ However, VMAT does not exhibit excellent PTV coverage in all cases. Kim et al. published a planning study comparing IMRT and VMAT plans to an existing 3D treatment plan for 10 SBBC patients.^9^ They reported that IMRT was superior to 3DCRT and VMAT in terms of PTV dose distribution, whereas VMAT exhibited the highest treatment efficiency.

All previous studies on SBBC patients have compared IMRT and VMAT under the single‐isocenter condition. In general, the dual‐isocenter technique is traditionally applied for the treatment of patients with breast cancer with supraclavicular and axillary lymph node involvement as well as mastectomy.^12^, ^13^, ^14^ Boman et al. investigated the feasibility of utilizing dual isocenters in VMAT planning for bilateral lymph node‐positive breast cancer.^12^ They reported that the dose parameters slightly favored the dual‐isocenter option over the single‐isocenter one. However, Amoush et al. and Banaei et al. recommended the use of a single isocenter in the case of breast cancer patients with supraclavicular nodes or mastectomy.^13^, ^14^

In this study, we assessed dosimetric differences between VMAT and IMRT using both single‐ and dual isocenters to treat SBBC without axillary lymph node involvement. The primary aim of this study was to suggest an efficient treatment option for patients with SBBC.

## 2 MATERIALS AND METHODS

### 2.1 Patient selection and contouring

A total of 20 patients with SBBC without lymph node involvement were retrospectively selected for this study. The mean age of the selected patients was 53 years (range, 42–72 years). A CT scan was performed during free breathing (FB) with a slice thickness of 5 mm using a Philips Brilliance CT Big Bore. All patients were immobilized with a breast board in the supine position with both arms raised to the forehead.

Following the ESTRO guidelines, contouring work for all targets and structures was outlined by the same oncology physician. The clinical target volume (CTVs) for bilateral breasts was defined to be a volume that includes the whole breast and the tumor bed, cropped 5 mm inside the body contour according to the Radiation Therapy Oncology Group contouring atlas group.^15^ The PTV was defined to be the union of the CTV with a treatment margin of 10 mm for superior–inferior, 7 mm for anterior–posterior, and 7 mm for left–right to allow set up uncertainties and account for breathing motion. The left lung, right lung, heart, and left anterior descending (LAD) artery were considered to be the OARs.

### 2.2 Dose prescription and planning techniques

The dose of 42.56 Gy in 16 fractions, instead of the historical standard regimen (50 Gy in 25 fractions), was prescribed to both PTVs as the Ontario‐Canadian trials.^16^ Appropriately administered hypofractionated RT has been reported in previous studies to be an effective and safe treatment, reducing the number of fractions as well as side effects such as acute pain, fatigue, and dermatitis.^17^ The plan objectives are listed in Table 1. The primary goal of planning was to ensure that at least 95% of the PTV received at least 95% of the prescribed dose to ensure adequate dose coverage of the target volume. In order to ensure PTV homogeneity, 108% of the prescribed dose was limited to less than 1% of the target volume. Dose constraints for OARs were established by modifying estimates of previous SBBC studies appropriately, due to the absence of a treatment protocol for SBBC.^17^, ^18^

**TABLE 1:** Dose constraints to target volume and organs at risk for planning synchronous bilateral breast cancer (SBBC)

Structure	Dose constraints
PTV^a^	*V*_46.0 Gy_ < 1%, *V*_40.5 Gy_ > 95%
Left lung	*D*_mean_ < 20 Gy, *V*_5 Gy_ < 80%, *V*_20 Gy_ < 30%, *V*_30 Gy_ < 20%
Right lung	*D*_mean_ < 20 Gy, *V*_5 Gy_ < 80%, *V*_20 Gy_ < 30%, *V*_30 Gy_ < 20%
Heart	*D*_mean_ < 10 Gy, *V*_20 Gy_ < 10%
Left anterior descending artery	*D*_mean_ < 24 Gy, *V*_30 Gy_ < 33%

^a^Planning target volume.

The treatment plans were generated using the Eclipse treatment planning system, and the dose distributions were calculated using the Acuros XB algorithm with a 0.25‐cm grid size. All plans used a photon optimizer for a 6‐MV photon beam of VitalBeam with the Millennium 120 MLC.

**FIGURE 1:**
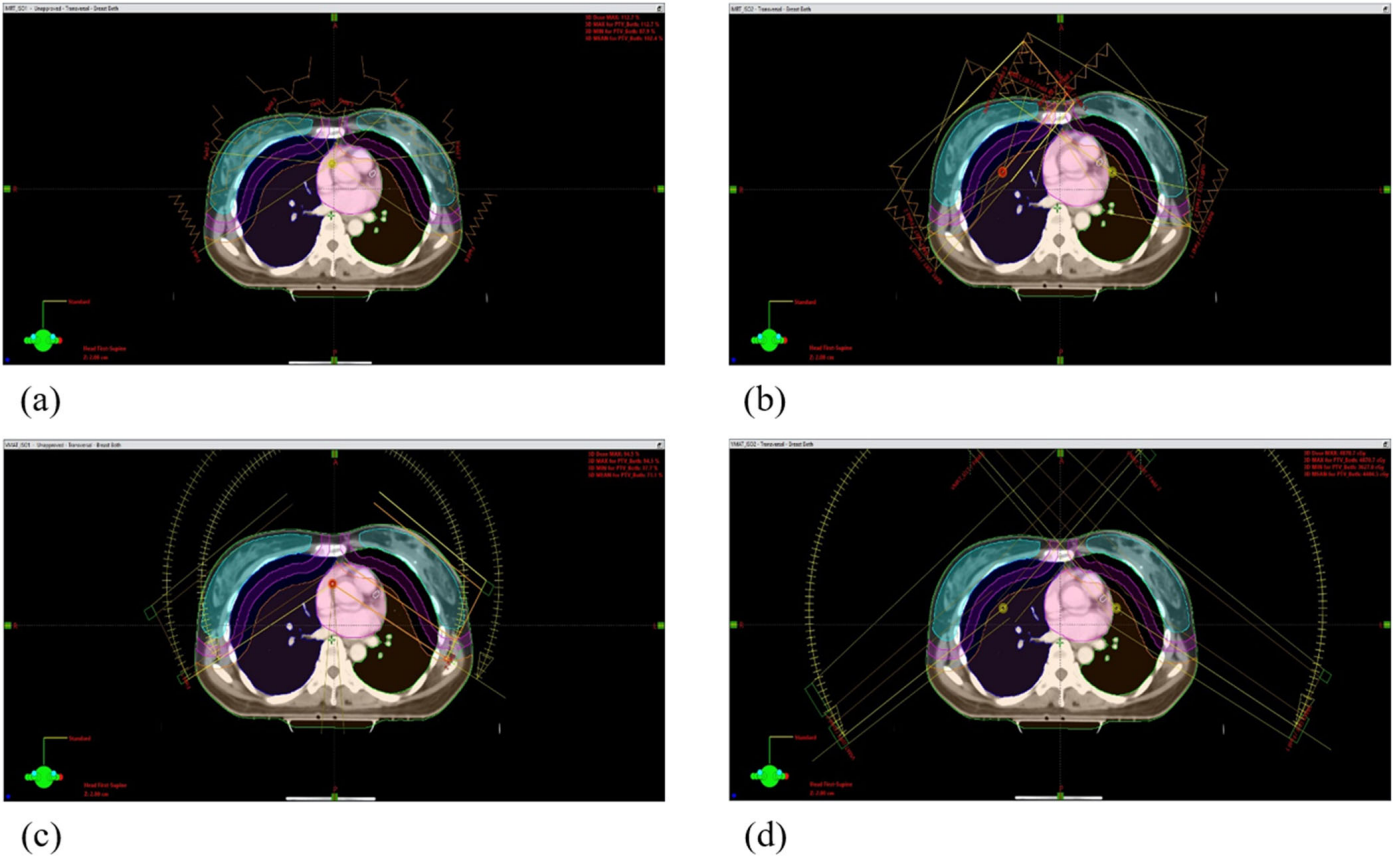
Beam arrangements of (a) IMRT_Iso1, (b) IMRT_Iso2, (c) VMAT_Iso1, and (d) VMAT_Iso2 according to the conditions of two delivery techniques and two isocenters

For each patient, four planning techniques (Figure 1) were implemented in this study using two delivery techniques under two isocenter conditions—IMRT using a single isocenter (IMRT_Iso1), VMAT using a single isocenter (VMAT_Iso1), IMRT using dual isocenters (IMRT_Iso2), and VMAT using dual isocenters (VMAT_Iso2). The IMRT_Iso1 plan was optimized with respect to a single isocenter located under the sternum. Eight beams were used at angles between 240° and 120°. Four of the eight beams were aligned at angles between 15° and 120° at intervals of 35°. The other beams were aligned at angles between 240° and 345° at intervals of 35°. The VMAT_Iso1 plan was also optimized with respect to the single isocenter used in the IMRT_Iso1 plan. Two partial arc beams were used, one with gantry start and stop angles of 240° and 120°, respectively, in the clockwise direction and the other with 120° and 240°, respectively, in the counterclockwise direction. The IMRT_Iso2 Plan was established with two isocenters, one for each breast. These two isocenters were located in the same vertical and longitudinal axes. Thus, the isocenter shift only needs to consider the lateral movement when moving from the first isocenter to the second. Four beams were applied to each breast, thus utilizing a total of eight beams. For the left breast, four tangential fields with gantry angles of 100°, 120°, 300°, and 320° were applied. For the right breast, four tangential fields with gantry angles of 40°, 60°, 220°, and 240° were used. The VMAT_Iso2 plan utilized the two isocenters used in IMRT_Iso2. For the left breast, two partial arcs between 120° and 310° (clockwise and counterclockwise) were used, and two partial arcs between 240° and 50° were used for the right breast.

### 2.3 Evaluation of dosimetric and radiobiological parameters

The dosimetric parameters of each plan were assessed based on cumulative dose–volume histograms (DVHs). For PTV, dosimetric parameters such as minimum dose (*D*_min_), mean dose (*D*_mean_), maximum dose (*D*_max_), and *V*_95%_ (percentage of the volume that received at least 95% of the prescribed dose) were analyzed. *V*_95%_ of the PTV was used as a measure of the target coverage. To evaluate plan quality, homogeneity index (HI), conformity index (CI), and conformation number (CN) were estimated in the PTV. HI was calculated using the following equation:

$$\mathrm{HI}=\frac{D_{2\%}-D_{98\%}}{D_{50\%}}$$

where *D*_2%_, *D*_98%_, and *D*_50%_ denote the doses corresponding to 2%, 98%, and 50% volume of the PTV, respectively.^19^ The HI values are inversely proportional to the degree of dose homogeneity in the PTV. CI was calculated based on the reference dose of the prescription dose to the PTV using the following equation:

$$\mathrm{CI}=\frac{V_{\mathrm{ref}}}{\mathrm{TV}}$$

where *V*_ref_ denotes the total volume of all areas surrounded by the reference isodose (reference isodose = 95%) on the body, and TV denotes the physical volume of the PTV. A CI equal to 1 corresponds to ideal conformation. The CI greater than 1 indicates that the irradiated volume is greater than the target volume and includes healthy tissues. If the CI is less than 1, the target volume is only partially irradiated.^20^

To evaluate conformity to the target dose and healthy tissue irradiation, CN was estimated using the following equation:

$$\mathrm{CN}=\frac{TV_{\mathrm{ref}}}{\mathrm{TV}}\times \frac{TV_{\mathrm{ref}}}{V_{\mathrm{ref}}}$$

where TV_ref_ denotes the PTV volume covered by the reference isodose. The first fraction of this equation defines the quality of target coverage, and the second fraction represents the volume of healthy tissue receiving a dose greater than or equal to the prescribed reference dose. The CN ranges from 0 to 1, where 1 is the ideal value.^21^

For OAR comparison, the following dosimetric parameters were evaluated under each plan—*V*_30 Gy_, *V*_20 Gy_, *V*_10 Gy_, and *V*_5 Gy_ (volumes that received 30, 20, 10, and 5 Gy doses), mean doses for left and right lung, mean dose and *V*_20 Gy_ for heart, and mean dose and *V*_20 Gy_ for LAD.

To investigate the radiobiological impact of the treatment on PTV and OARs using a MATLAB software‐based program, the equivalent uniform dose (EUD^2Gy^) corresponding to an equivalent dose related to a reference of 2 Gy per fraction, the tumor control probability (TCP), and the normal tissue complication probability (NTCP) were calculated.^10^, ^22^, ^23^, ^24^, ^25^, ^26^, ^27^ Table 2 lists the radiobiological parameters used during SBBC irradiation in the four treatment regimens using the EUD‐based model.

**TABLE 2:** The radiobiological parameters used for synchronous bilateral breast cancer (SBBC) irradiation

Organ	*a*	γ_50_	TD_50_ (Gy)	TCP_50_ (Gy)	Alpha–beta ratio
PTV^a^	−7.2	2		28	4
Lung	1	2	24.5		3.9
Heart	3	3	48		2

^a^Planning target volume.

To evaluate treatment efficiency, delivery parameters, monitor units (MUs), and delivery time were recorded in each of the four treatment regimens.

## 3 RESULTS

### 3.1 Dosimetric parameters for target volume

**FIGURE 2:**
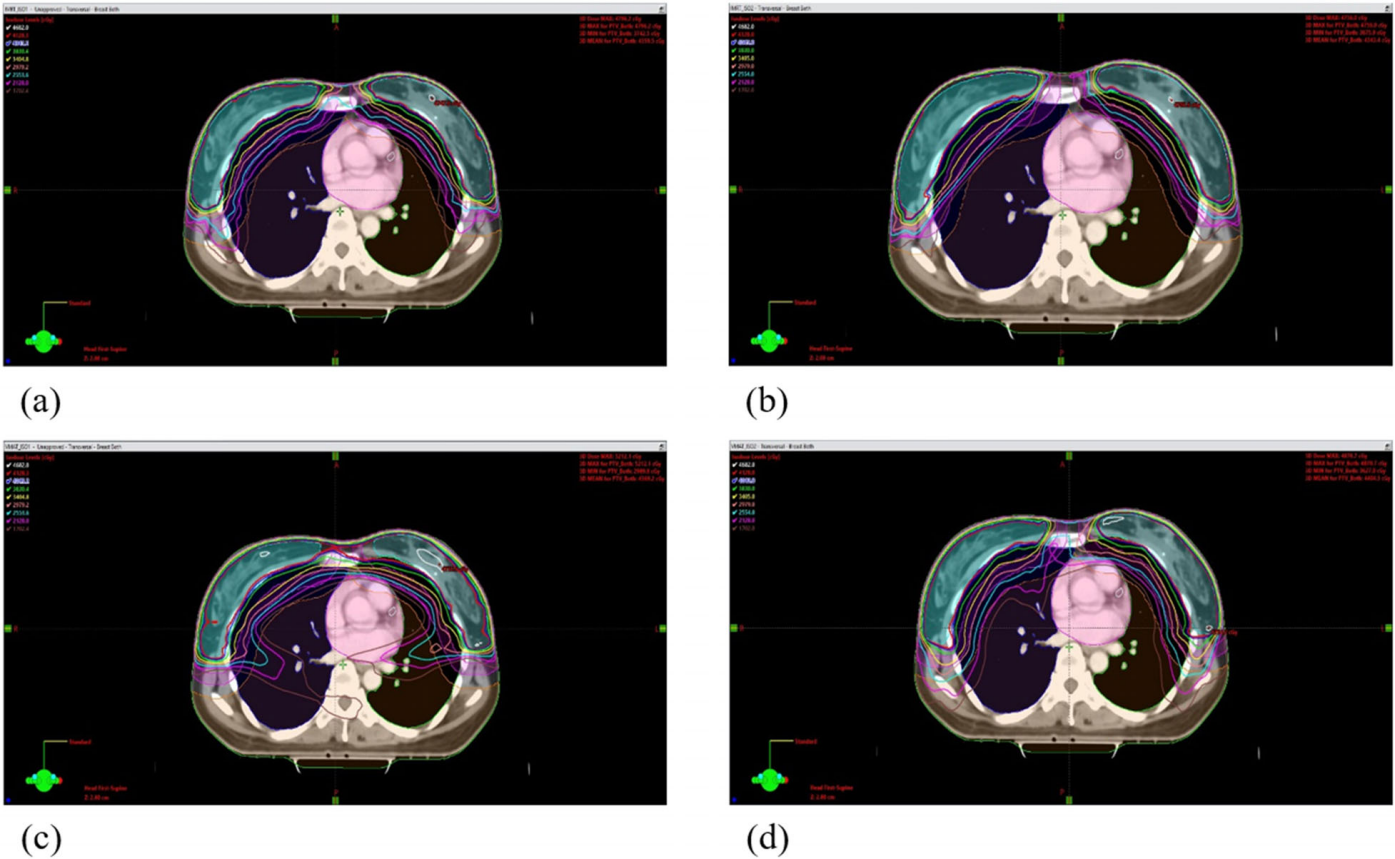
Dose distributions on an axial view of (a) IMRT_Iso1, (b) IMRT_Iso2, (c) VMAT_Iso1, and (d) VMAT_Iso2 plans for one patient case

**FIGURE 3:**
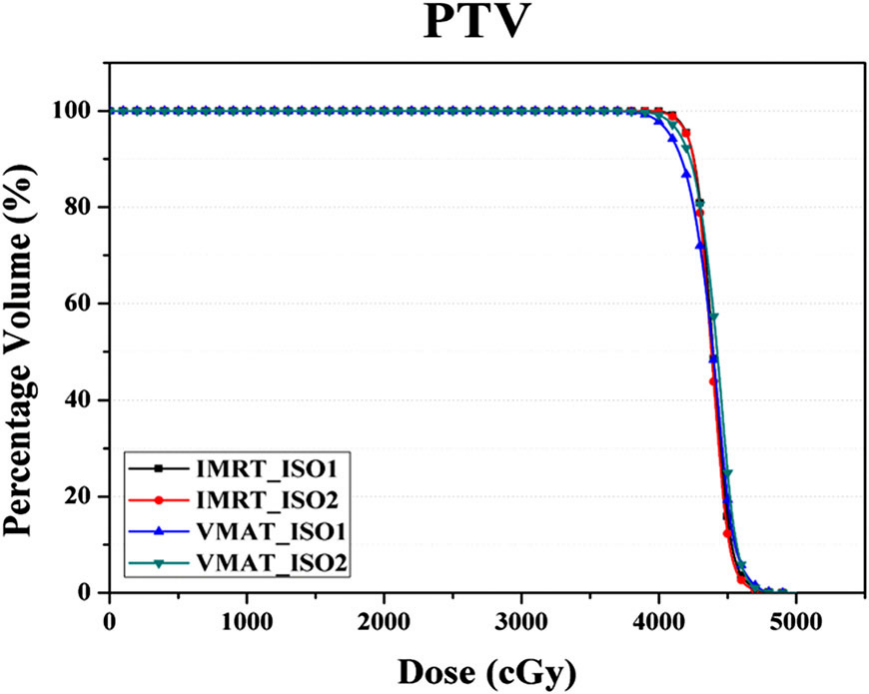
Mean dose–volume histogram (DVH) of planning target volume (PTV) for four treatment regimens of 20 synchronous bilateral breast cancer (SBBC) patients

**FIGURE 4:**
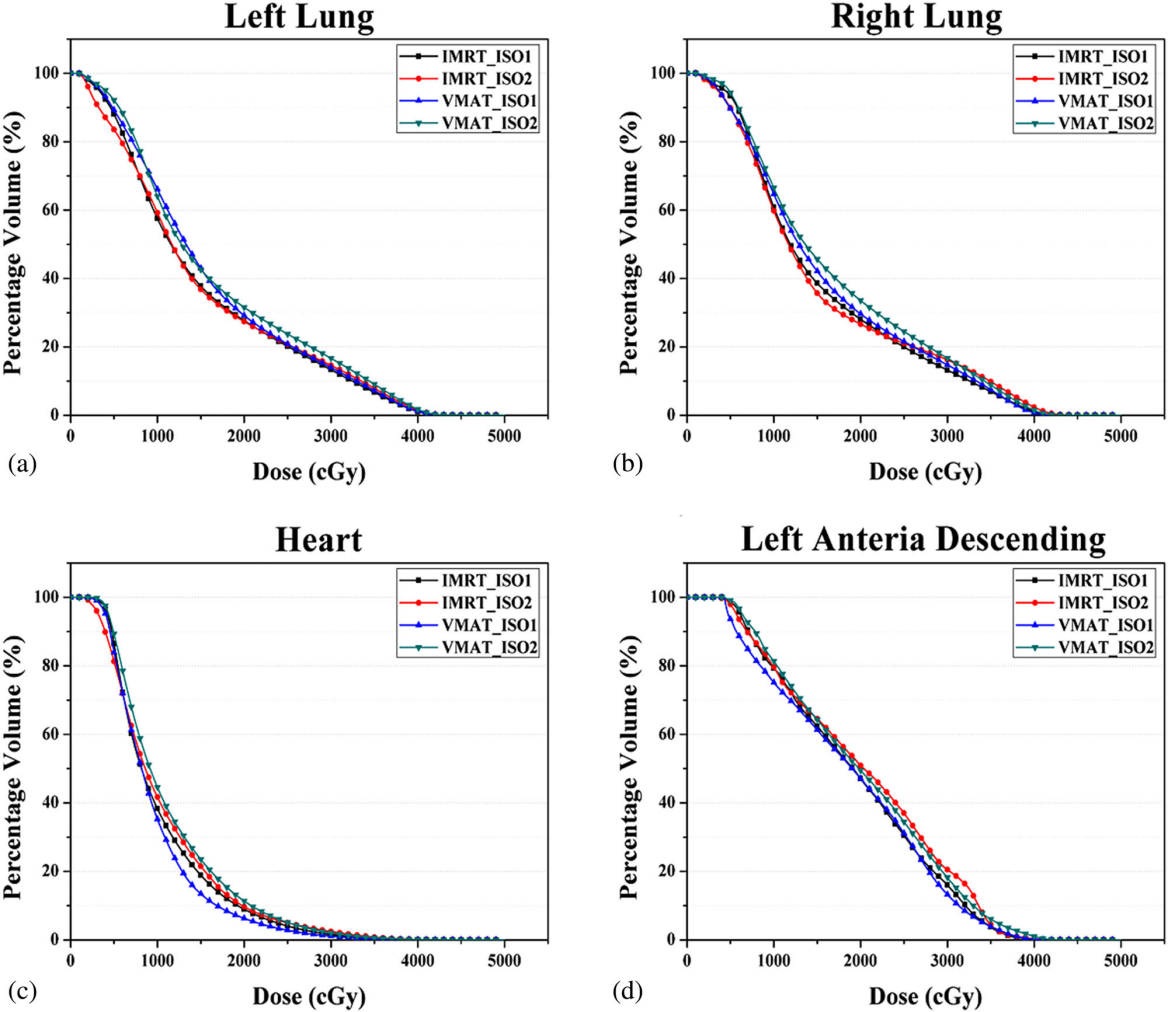
Mean dose–volume histogram (DVH) of (a) left lung, (b) right lung, (c) heart, and (d) left anterior descending (LAD) for four treatment regimens of 20 synchronous bilateral breast cancer (SBBC) patients

Figure 2 depicts an example of dose distributions corresponding to the four treatment regimens—(a) IMRT_Iso1, (b) IMRT_Iso2, (c) VMAT_Iso1, and (d) VMAT_Iso2. Figures 3 and 4 present examples of mean DVHs for PTV and OARs in the four treatment plans using two delivery techniques under two isocenter conditions. The mean and standard deviation of the dosimetric parameters corresponding to the PTV for the cohort of 20 patients are summarized in Table 3.

**TABLE 3:** The comparison of dosimetric parameters to planning target volume (PTV) in four treatment regimens according to the conditions of two delivery techniques and two isocenters

	Index	IMRT_Iso1	IMRT_Iso2	VMAT_Iso1	VMAT_Iso2
PTV	*D*_max_ (Gy)	47.89 ± 0.61	48.28 ± 1.04	48.88 ± 1.01	48.39 ± 0.57
	*D*_mean_ (Gy)	43.95 ± 0.29	43.82 ± 0.21	43.75 ± 0.19	44.09 ± 0.16
	*D*_min_ (Gy)	36.61 ± 1.56	34.68 ± 3.67	32.84 ± 2.30	34.42 ± 1.72
	*V*_95%_ (%)	99.70 ± 0.24	99.53 ± 0.56	97.03 ± 1.74	98.78 ± 0.85
	HI^a^	0.11 ± 0.01	0.11 ± 0.01	0.15 ± 0.02	0.13 ± 0.01
	CI^b^	1.00 ± 0.00	1.00 ± 0.01	0.97 ± 0.02	0.99 ± 0.01
	CN^c^	0.84 ± 0.03	0.83 ± 0.03	0.84 ± 0.04	0.84 ± 0.04

^a^Homogeneity index.

^b^Conformity index.

^c^Conformation number.

In terms of *D*_max_, *D*_mean_, *D*_min_, and *V*_95%_ to the PTV, slight differences were observed among the four treatment plans corresponding to each patient. IMRT_Iso1 exhibited the lowest *D*_max_ value (47.89 ± 0.61 Gy), whereas that of VMAT_Iso1 (48.88 ± 1.01 Gy) was much higher than those of the other techniques. Moreover, the dosage of the VMAT_Iso1 technique was ∼2.0% higher than that of IMRT_Iso1, and 1.2% and 1.0% higher than those of IMRT_Iso2 and VMAT_Iso2, respectively. VMAT_Iso1 exhibited the lowest *D*_mean_ (43.75 ± 0.19 Gy), whereas VMAT_Iso2 recorded the highest (44.09 ± 0.16 Gy). Among the four treatment plans, slight differences (∼1%) were observed in *D*_mean_ and PTV. VMAT_Iso1 exhibited the lowest *D*_min_ (32.84 ± 2.30 Gy), whereas IMRT_Iso1 exhibited the highest (36.61 ± 1.56 Gy). Among *D*_max_, *D*_mean_, and *D*_min_, the largest difference between the different treatment plans was manifested in terms of *D*_min_. Further, IMRT_Iso1 (99.70 ± 0.24) exhibited higher *V*_95%_ that the other techniques (for IMRT_Iso2, *V*_95%_ = 99.53 ± 0.26; for VMAT_Iso1, *V*_95%_ = 97.03 ± 1.74; and for VMRT_Iso2, *V*_95%_ = 98.78 ± 0.85). Thus, in all four plans considered in this study, at least 97% of the PTV received 95% of the prescription dose. IMRT_Iso1 covered a volume ∼2.68% higher than that of VMAT_Iso1 and 0.92% higher than that of VMAT_Iso2.

In terms of parameters, such as HI, CI, and CN to the PTV, there was no significant difference between the different plans. HI values close to 0 indicate better homogeneity over the PTV. The HI was 0.11 ± 0.01, 0.11 ± 0.01, 0.15 ± 0.02, and 0.13 ± 0.01 for IMAT_Iso1, IMAT_Iso2, VMAT_Iso1, and VMAT_Iso2, respectively. IMRT_Iso1 and IMRT_Iso2 were more homogeneous than VMAT_Iso1 and VMAT_Iso2. On the other hand, CI values close to 1 correspond to ideal conformation over the PTV. IMAT_Iso1 (1.00 ± 0.00) and IMRT_Iso2 (1.00 ± 0.01) exhibited the highest CI values, followed by VMAT_Iso1 (0.97 ± 0.02) and VMAT_Iso2 (0.99 ± 0.01). All treatment regimens had good CI close to 1. The CNs of all treatment regimens were similar with no significant difference.

### 3.2 Dosimetric and radiobiological parameters related to OARs

Table 4 presents a statistical comparison of the dosimetric parameters corresponding to OARs under the four treatment regimens. Data summarized in Table 4 represent the mean and standard deviation computed over the cohort of 20 patients. For OARs, the *D*_mean_ (15.08 ± 0.73 Gy), *V*_5 Gy_ (72.77% ± 3.77%), and *V*_20 Gy_ (27.41% ± 3.86%) of the left lung were lower under IMRT_Iso2 than under IMRT_Iso1, VMAT_Iso1, and VMAT_Iso2. These parameters under IMRT_Iso2 were significantly different from those under VMAT_Iso1 and VMAT_Iso2, but not significantly different from those under IMRT_Iso1. In addition, the lowest values of *V*_10 Gy_ and *V*_30 Gy_ were observed corresponding to IMRT_Iso1 and VMAT_Iso2, respectively. *D*_mean_ of IMRT_Iso1 (15.38 ± 0.37 Gy) for the right lung was the lowest, followed by IMRT_Iso2 (15.49 ± 0.72 Gy), VMAT_Iso1 (15.87 ± 1.45 Gy), and VMAT_Iso2 (16.86 ± 1.37 Gy). In the cases of *V*_5 Gy_, *V*_10 Gy_, and *V*_20 Gy_, IMRT_Iso2 exhibited lower values than IMRT_Iso1, VMAT_Iso1, and VMAT_Iso2. VMAT_Iso2 exhibited the lowest *V*_30 Gy_, and IMRT_Iso1 exhibited the highest. *D*_mean_ for the heart was the lowest under IMRT_Iso2 (9.05 ± 0.59 Gy) and highest under VMAT_Iso2 (11.08 ± 0.84 Gy). In addition, VMAT_Iso1 (6.30 ± 4.15 Gy) exhibited the lowest *V*_20 Gy_, and VMAT_Iso2 (11.34 ± 5.36 Gy) exhibited the highest. The *D*_mean_ and *V*_20 Gy_ for the LAD were the lowest under VMAT_Iso1 (18.01 ± 4.62 Gy and 13.48 ± 13.14 Gy, respectively) and the highest under IMRT_Iso1 (19.31 ± 4.36 Gy and 16.33 ± 18.17 Gy, respectively).

**TABLE 4:** The comparison of dosimetric parameters to organs at risk (OARs) in four treatment regimens

Organs	Index	IMRT_Iso1	IMRT_Iso2	VMAT_Iso1	VMAT_Iso2
Lt lung	*D*_mean_ (Gy)	15.12 ± 0.77	15.08 ± 0.73	16.00 ± 1.16	16.59 ± 1.05
	*V*_5 Gy_ (%)	77.40 ± 4.71	72.77 ± 3.77	88.73 ± 5.80	91.40 ± 3.82
	*V*_10 Gy_ (%)	57.55 ± 6.51	59.23 ± 4.79	65.85 ± 7.50	64.10 ± 5.11
	*V*_20 Gy_ (%)	27.67 ± 3.54	27.41 ± 3.86	28.64 ± 4.13	31.35 ± 3.60
	*V*_30 Gy_ (%)	16.67 ± 2.99	14.58 ± 3.22	13.72 ± 2.15	13.32 ± 2.25
Rt lung	*D*_mean_ (Gy)	15.38 ± 0.37	15.49 ± 0.72	15.87 ± 1.45	16.86 ± 1.37
	*V*_5 Gy_ (%)	79.62 ± 5.47	79.07 ± 2.82	82.74 ± 2.42	83.88 ± 3.28
	*V*_10 Gy_ (%)	60.87 ± 6.43	59.79 ± 5.84	64.71 ± 7.40	66.54 ± 5.51
	*V*_20 Gy_ (%)	28.07 ± 3.27	26.66 ± 4.18	29.63 ± 4.85	30.55 ± 5.38
	*V*_30 Gy_ (%)	16.70 ± 3.59	16.22 ± 3.53	14.73 ± 3.23	13.23 ± 2.47
Heart	*D*_mean_ (Gy)	10.34 ± 2.47	9.05 ± 0.59	9.85 ± 1.13	11.08 ± 0.84
	*V*_20 Gy_ (%)	8.96 ± 6.82	8.69 ± 7.29	6.30 ± 4.15	11.34 ± 5.36
LAD^a^	*D*_mean_ (Gy)	19.31 ± 4.36	18.25 ± 5.22	18.01 ± 4.62	18.17 ± 4.26
	*V*_20 Gy_ (%)	16.33 ± 18.17	15.56 ± 20.18	13.48 ± 13.14	16.09 ± 15.41

^a^Left anterior descending.

### 3.3 Radiobiological parameters

The values of EUD^2Gy^ and TCP over the PTV and EUD^2Gy^ and NTCP over normal tissues are presented in Table 5. In general, there was no meaningful difference between EUD^2Gy^ (<0.5 Gy) and TCP (<0.1%) in the PTV under IMRT and VMAT with both isocenter conditions. As presented in Table 5, the EUD^2Gy^ and NTCP of the left and right lungs were slightly higher under VMAT than under IMRT. On the other hand, the EUD^2Gy^ for the heart was lower under VMAT_Iso1 than the other treatment regimens. The calculated NTCP for the heart was zero for all four treatment regimens.

**TABLE 5:** The tumor control probability (TCP) and the equivalent uniform dose (EUD) value for planning target volume (PTV) and the normal tissue complication probability (NTCP) and EUD values for organs at risk (OARs)

Organs	Index	IMRT_Iso1	IMRT_Iso2	VMAT_Iso1	VMAT_Iso2
PTV	EUD^2Gy^ (Gy)	49.17 ± 0.45	48.97 ± 0.31	48.76 ± 0.46	49.21 ± 0.32
	TCP (%)	98.90 ± 0.08	98.87 ± 0.06	98.79 ± 0.09	98.91 ± 0.06
Lt lung	EUD^2Gy^ (Gy)	13.55 ± 0.76	13.62 ± 0.72	14.44 ± 1.72	15.09 ± 2.33
	NTCP (%)	0.76 ± 0.37	0.84 ± 0.49	1.20 ± 1.18	1.09 ± 1.40
Rt lung	EUD^2Gy^ (Gy)	13.94 ± 0.47	14.13 ± 0.80	14.47 ± 1.44	15.52 ± 1.43
	NTCP (%)	1.12 ± 0.31	1.33 ± 0.69	1.93 ± 2.06	3.10 ± 2.18
Heart	EUD^2Gy^ (Gy)	11.63 ± 2.61	12.64 ± 2.75	10.61 ± 2.22	12.85 ± 2.21
	NTCP (%)	0.00 ± 0.00	0.00 ± 0.00	0.00 ± 0.00	0.00 ± 0.00

### 3.4 Delivery parameters

Table 6 records the delivery parameters used to investigate treatment efficiency of the four treatment regimens. Of the four delivery techniques, IMRT_Iso2 required the highest number of MUs, and VMAT_Iso1 required the fewest. VMAT_Iso1 had 103.8% fewer MUs than IMRT_Iso2. On the other hand, the MU of VMAT_Iso1 was 22% less than that of VMAT_Iso2. The required delivery time, indicating the beam‐on‐time, was also the lowest under VMAT_Iso1. IMRT_Iso2 required the longest beam delivery time of ∼2.6 min on average. VMAT_Iso1 exhibited a 45.9% shorter delivery time than IMRT_Iso2, which, in turn, exhibited a delivery time that was 22.1% shorter than that of IMRT_Iso1 and 20.9% shorter than that of VMAT_Iso2.

**TABLE 6:** The comparison of the delivery parameters for the four treatment regimens

Index	IMRT_Iso1	IMRT_Iso2	VMAT_Iso1	VMAT_Iso2
MU^a^	2016 ± 63	2469 ± 86	1211 ± 23	1483 ± 54
Delivery time (s)	210 ± 39	251 ± 51	172 ± 11	208 ± 28

^a^Monitor unit.

## 4 DISCUSSION

Dosimetrical parameters of various techniques based on 3D‐CRT, IMRT, and VMAT for SBBC patients have been evaluated in many previous studies.^5^, ^6^, ^9^ However, few studies have compared the dosimetric and radiobiological parameters of IMRT and VMAT using two isocenters for SBBC. We compared IMRT and VMAT plans under two isocenter conditions for SBBC treatment during the course of a hypofractionated RT comprising 16 fractions.

In terms of PTV coverage, the average differences between the four treatment plans were small, but IMRT_iso1 exhibited better performance corresponding to each patient than the other three techniques. There was no significant difference in the values of parameters such as HI, CI, and CN, thus indicating similar plan quality. As the same objective function was used for the target constraints with high priority in both IMRT and VMAT planning, there was no difference in dosimetric parameters related to PTV for the four treatment regimens. However, Kim et al. reported that IMRT performs better than VMAT when both are implemented using a single isocenter.^9^ Further, they attributed the difference to the MONACO TPS used in their study. In contrast, Nicolini et al. reported that VMAT performs better than IMRT in terms of target coverage and homogeneity.^8^ Contrary to the suggestion of some studies that a dual‐isocentric solution may be necessary for very large bilateral breasts and bilateral lymph node‐positive breasts, this study shows that there is no significant difference in PTV coverage between single‐ and dual‐isocenter cases for SSBC patients without lymph nodes.^3^, ^13^

Concerning all OARs, most plans satisfied the dose constraints of dosimetric parameters presented in Table 1, except the cases of *V*_5 Gy_ of both lungs and *D*_mean_ of the heart in VMAT_Iso1 and VMAT_Iso2m, respectively. High sparing of most OARs was achieved using IMRT_Iso2, with the exception of *V*_20 Gy_ of the heart and *D*_mean_ and *V*_20 Gy_ of the LAD. There was little difference in OAR sparing between IMRT_Iso1 and IMRT_Iso2. IMRT_Iso2 also exhibited excellent *D*_mean_ of the heart, which has been used as a reference in cardiotoxicity studies. Darby et al. found a linear relationship between the *D*_mean_ of the heart and the incidence of ischemic heart disease, which was reported to increase by 7.4% per Gy of *D*_mean_ of the heart.^28^ There was no significant difference in OAR sparing in the case of IMRT implemented using one and two isocenters, but single‐isocentric VMAT exhibited better dosimetric parameters than the dual‐isocentric one at low doses. IMRT outperformed VMAT in OAR sparing in all cases except for the high‐dose volume in both lungs. Further, VMAT exhibited a large spread in low‐dose and intermediate volumes in both lungs. This can be attributed to the delivery of radiation in arc form in VMAT within the set angular range to cover the huge C‐shaped target volume surrounding both lungs, rather than delivery only at the angle used, as in the case of IMRT. Previous studies have reported that the low‐dose spillage around the target volume is greater in VMAT than in IMRT.^29^, ^30^ According to a previous study by Boman et al., the use of dual isocenters in VMAT reduces the average dose to the bilateral lungs and heart compared to the use of a single isocenter.^12^ However, our study could not find a dosimetric improvement by using dual isocenters in IMRT and VMAT in SBBC patients.

Radiobiological analysis revealed that EUD^2Gy^ and TCP for PTV were roughly similar corresponding to all regimens. EUD^2Gy^ and NTCP of both lungs were slightly lower under IMRT than under VMAT. In addition, the treatment plans using a single isocenter exhibited slightly lower EUD^2Gy^ and NTCP than those using dual isocenters within the same delivery technique. As a result, it is expected that the lowest incidence of pneumonitis was considered to be lung toxicity in SBBC patients treated using IMRT_Iso1. On the other hand, NTCP of the heart was 0% under all regimens. The occurrence of pericarditis, which is considered to be an endpoint of heart toxicity, could be ruled out completely.

The VMAT technique exhibited some advantages over IMRT in terms of MU and delivery time, as indicated in Table 6. However, IMRT_Iso1, which requires a relatively large number of MUs, exhibited a 22% longer delivery time than VMRT_Iso1, whose delivery time was shorter than expected. This can be attributed to the act that the IMRT treatment beam is fixed and delivered at a constant rate of 600 MU/min, whereas that of VMAT is delivered at a rate that varies with the gantry rotation. The MU and delivery time of both techniques increased with an increase in the number of beams and isocenters. This proves that an increase in the number of beams and isocenters does not always increase the treatment efficiency of IMRT and VMAT plans in SBBC patients. In addition, the delivery time considered in this study only takes into account the pure beam on time, which does not include the patient setup and pretreatment imaging stages that are required to verify patient positioning. Thus, the actual treatment time is expected to increase significantly in clinical practice, especially for treatment techniques using dual isocenters. While using a dual‐isocenter technique, couch movement treatment typically requires a therapist to enter the treatment room and manually perform the lateral shift, necessitating additional work and time during the treatment session. In addition, the use of dual isocenters in SBBC treatment may introduce inaccuracies into the setup stage, for example, the patient's lateral movement or couch shift when moving from the first isocenter to the second. Moreover, previous studies have introduced the possibility of large errors (>5 mm) when moving from the first treatment isocenter to the second.^12^ Therefore, the use of a single isocenter in the medial location under the sternum used in this study will be advantageous in reducing setup error and may reduce the risk of possible treatment head collision to the couch or the patient in rotational treatments.

A limitation of this study is that four treatment regimens for SBBC patients were conducted while the patients were in the FB state. In general, respiratory‐gated radiotherapy and deep inspiration breath hold (DIBH) are known to have clinical and dosimetric benefits in breast RT.^31^, ^32^, ^33^, ^34^ Bruzzaniti et al. reported that the execution of DIBH during RT of breast cancer reduces both the mean and maximum doses to the heart in a statistically significant way by reducing the heart volume included in the irradiation fields.^31^ In addition, Sixel et al. concluded that the risk of radiation pneumonitis does not increase because of DIBH beyond that caused by standard clinical practice with regular tangents and quiet respiration.^34^ As such, IMRT_Iso1 using DIBH for SBBC is expected to further reduce the radiation risk to the lungs and heart. However, in this study, respiration management was not considered. In the future, we intend to conduct a study on the use of DIBH to further reduce the radiation risk of OARs in SBBC patients.

## 5 CONCLUSION

Compared to other treatment regimens for patients with SBBC, IMRT_Iso1 exhibited almost similar target coverage and a certain degree of dosimetric improvement in OAR sparing. Moreover, it also exhibited radiobiological improvement over the others, although the difference in performance was not significant. Although it significantly increased MU compared to VMAT_Iso1, which performed the best in terms of delivery efficiency, the delivery time only increased by 22% because of the use of a constant rate of delivery of the dose. Therefore, in conclusion, IMRT_Iso1, the complete treatment of which can be completed using a single setup, is the most effective method for treating SBBC.

## AUTHOR CONTRIBUTIONS

Study concept and design: Jin‐Beom Chung, data acquisition: Sang‐Won Kang, Seonghee Kang, Boram Lee, Woong Cho, Dong‐Suk Shin, and Jin‐Young Kim, data analysis and interpretation: Keun‐Yong Eom, Changhoon Song, Bum‐Sup Jang, In Ah Kim, and Jae‐Sung Kim. Manuscript preparation and editing: Sang‐Won Kang, Seonghee Kang, and Jin‐Beom Chung. All authors contributed to the article and approved the submitted version.
